# An extension of the validation cohort of the Dutch Early-Stage Melanoma (D-ESMEL) study for stage-specific analyses

**DOI:** 10.1007/s10654-025-01337-3

**Published:** 2026-01-12

**Authors:** Catherine Zhou, Antien L. Mooyaart, Nikita Hulscher, Thamila Kerkour, Jasper Ouwerkerk, Marieke W. J. Louwman, Marlies Wakkee, Yunlei Li, Quirinus J. M. Voorham, Annette Bruggink, Tamar E. C. Nijsten, Loes M. Hollestein

**Affiliations:** 1 Department of Dermatology, Erasmus MC Cancer Institute, Rotterdam, The Netherlands; 2 Department of Pathology, Erasmus MC Cancer Institute, Rotterdam, The Netherlands; 3 Department of Pathology & Clinical Bioinformatics, Erasmus MC Cancer Institute, University Medical Center Rotterdam, Rotterdam, The Netherlands; 4 Department of Research and Development, Netherlands Comprehensive Cancer Organization, Utrecht, The Netherlands; 5 Dutch Nationwide Pathology Databank (Palga Foundation), Houten, The Netherlands

**Keywords:** Nested case-control study, Melanoma, Prognostic model, Stage II

## Abstract

There is a high need for accurate prognostic models among stage II melanoma to determine who may benefit from (neo)adjuvant systemic therapy. The Dutch Early- Stage Melanoma (D-ESMEL) study was designed to identify new prognostic features in a population-based sample of stage I/II melanoma patients in addition to American Joint Committee of Cancer (AJCC) staging. The validation cohort of the D-ESMEL study employs a nested case-control design. Initially, controls were randomly sampled to develop prognostic that included both known and new prognostic factors to assess the additive value of new prognostic factors. As a consequence, most controls had a very thin melanoma (<1.0 mm) while most cases had a thicker melanoma (>2.0 mm). This resulted in insufficient variability and high weights for stage II controls when applying weighted analyses in absolute risk prediction models. Therefore, randomly sampled controls were re-matched on AJCC stage (stage IA, IB, IIA, IIB, IIC), and new stage-matched controls were collected for cases who could not be rematched. The original D-ESMEL validation cohort included 5,815 stage I/II melanoma patients, of whom 154 developed distant metastasis (cases). 98/154 Cases were stage II and only 24 stage II controls were included, while the stage-matched design now includes 153 stage-matched case-control sets of which 97 stage II cases and 97 stage II controls derived from a population-based cohort of 5,785 stage I/II patients. The updated design increased the biological variability among stage II controls, balanced weights in weighted analyses and thereby facilitating reliable subgroup analyses in this clinically important subgroup.

## Introduction

The Dutch Early-Stage Melanoma (D-ESMEL) study was designed to identify new prognostic factors in American Joint Committee of Cancer (AJCC) stage I/II melanoma, because 40–60% of patients who die due to melanoma, were initially diagnosed with stage I/II [1, 2]. In the discovery set of the D-ESMEL study, cases and controls were exactly matched on known strong prognostic variables to identify new independent features. The validation cohort of the D-ESMEL study followed a nested case-control study, based on a population-based sample of all stage I/II patients from the Dutch population. The validation cohort was designed to build prognostic models that included both stage and newly identified prognostic factors to assess their added value. Therefore, controls were randomly selected. However, the majority of melanomas that are diagnosed in Western populations are thin, due to overdiagnosis, increased awareness and shifts in diagnostic criteria [4, 5]. As a result, the large amount of stage IA (very thin) melanomas in the source population and random sampling of controls led to the inclusion of controls that were mainly stage IA, while cases (patients who developed metastasis) were mostly diagnosed with thicker melanomas (stage IB to IIC). The low amount of stage II controls had methodological disadvantages. Scarcity of stage II controls resulted in low statistical power to perform stage-specific subgroup analyses, making it impossible to detect small differences in prognostic features within stage II. Second, in a nested case-control study, absolute risks in the population can be calculated by weighting the selected controls based on their occurrence in the total population [6]. The low amount of stage II controls resulted in disproportionately high weights per control (e.g. a single stage IIC control accounted for all 150 stage IIC patients without metastasis in the source cohort). Therefore, the analyses do not represent the biological variability in the total population.

As an improved prognostic model for stage II melanoma is of importance for clinical practice to guide adjuvant treatment decisions, we collected stage-matched controls to enable stage-specific subgroup analyses. Here, we describe this extension of the validation cohort of the Dutch Early-Stage Melanoma (D-ESMEL) study.

## Methods

### Summary of the original D-ESMEL study design

Data sources, study designs and tumor samples processing of the D-ESMEL study were described previously [3]. In summary, data from the Netherlands Cancer Registry (NCR) were linked to the Dutch Nationwide Pathology Databank (Palga) to obtain clinical data on all stage I/II cutaneous melanoma patients and retrieve the pathology reports and formalin-fixed paraffin-embedded (FFPE) tumor material of selected patients.

The discovery set was a matched case-control study and included 221 patients with stage I/II melanoma who developed distant metastasis during follow-up (cases) and 221 patients who did not develop distant metastasis (controls). Controls were matched to cases on follow-up time, age, sex, Breslow thickness and ulceration.

The validation cohort employed a nested case-control design and was based on all stage I/II cutaneous melanoma patients from the Netherlands between 2016 and 2020. The source cohort included 5,815 stage I/II melanoma patients. FFPE tumor samples were retrieved from 154 cases and 154 randomly selected controls, who had at least the same amount of follow-up time as their matched case.

### Selection of new stage-matched controls

We matched new controls to cases in the validation cohort based on follow-up time and AJCC stage in five categories (IA, IB, IIA, IIB, IIC). This stage-based matching approach offered two key advantages compared to exact matching on Breslow thickness and ulceration: it enabled assessment of the additional value of new prognostic factors independent of stage, reflecting clinical practice, and it facilitated the matching procedure by providing more potential matches per case.

First, we rematched the previously selected random controls to cases based on AJCC stage, sample type (punch/shave biopsy or elliptical excision) and follow-up time. For the remaining cases, new controls from the source population (*N* = 14,198 stage I/II melanomas) were selected. The pathology reports of the new controls were manually reviewed to confirm eligibility (e.g. a clear pathological description of the primary melanoma and the absence of distant metastasis documentation). Initially, FFPE material of three new potential controls per case were requested to ensure that at least one suitable control would be available for tissue processing, but only one control per case was selected for further tissue processing. Tumor samples processing remains the same as in the original D-ESMEL study [3]. After finalizing the extended dataset of all case-control sets, the size of the source population was recalculated based on the proportion of included cases to maintain a comparable event rate in this source cohort [3].

### Statistical analyses

Here we present descriptive statistics of the original D-ESMEL validation cohort and the extended D-ESMEL study, including the stage-matched controls. Differences between cases and controls were assessed using the McNemar’s test or the McNemar-Bowker test for > 2 categories for categorical variables. For continues variables the Wilcoxon signed-rank test was used to take the paired nature of the data into account. Statistical analyses were conducted using SAS^®^ (9.4 M8) and R studio (version 4.3.3.).

## Results

The original D-ESMEL validation cohort included 5,815 stage I/II melanoma patients, of whom 154 developed distant metastasis (cases) (Table 1). The controls were randomly selected from the source cohort, resulting in a predominance of stage IA melanoma (89/154, 58%). While 25% of all cases (38/154) had a stage IIC primary melanoma, only one control with stage IIC primary melanoma was included. Of all 154 cases, 77 could be rematched to a randomly selected control based on AJCC stage and follow-up time. For the remaining 77 cases, new controls were selected from the source cohort. For 1 stage IIC case, FFPE material of a stage-matched control was not received. In total, 153 cases could be matched to a control based on stage (i.e. either rematched randomly selected control or a newly selected control). These 153 cases originated from a recalculated source cohort of 5,785 patients in order to maintain the same event rate.

**Table 1:** Clinical characteristics of the original and extended validation cohort

	The D-ESMEL validation cohort							
	Original design Nested case-control study with randomly selected controls				Extended design Nested case-control study with stage-matched controls			
	Total	All cases from source cohort	Randomly selected controls	*p*-value (cases vs. randomly matched controls)	Total	All cases from source cohort	Stage-matched controls	*p*-value (cases vs. stage-matched controls)
**Characteristic**	(*n* = 5.815)	(*n* = 154)	(*n* = 154)		(*n* = 5.785)	(*n* = 153)	(*n* = 153)	
**Sex (*n*** **(%))**								
Male	2837 (49)	83 (54)	65 (42)	0.05	2783 (48)	83 (54)	69 (45)	0.13
Female	2978 (51)	71 (46)	89 (58)		3002 (52)	70 (45)	84 (55)	
**Age**,** years (median (IQR))**	62 (51–73)	68 (55–77)	63 (53–72)	0.01	63 (51–73)	68 (55–76)	70 (56–79)	0.48
**Breslow thickness**,** mm (median (IQR**,** min-max range))**	0.8 (0.5–1.4, 0.1–60.0)	2.7 (1.3–4.6, 0.3–32.0)	0.8 (0.5–1.3, 0.2–14.5)	< 0.001	0.8 (0.5–1.4, 0.1–61.0)	2.6 (1.3–4.6, 0.3–32.0)	2.7 (1.2–4.9, 0.2–61.0)	0.61
**Breslow thickness**,** mm (*n*** **(%))**								
*≤* 1.0	3744 (64)	25 (16)	95 (62)	< 0.001	3710 (64)	25 (16)	24 (16)	0.77
> 1.0–2.0	1120 (19)	37 (24)	38 (25)		1087 (19)	37 (24)	38 (25)	
> 2.0–4.0	559 (10)	40 (26)	14 (9)		568 (10)	40 (26)	39 (26)	
> 4.0	384 (7)	52 (34)	7 (5)		401 (7)	51 (33)	52 (34)	
**Ulcerated (*n*** **(%))**	533 (9)	68 (44)	12 (8)	< 0.001		67 (44)	61 (40)	0.36
**Stage (*n*** **(%))**								
IA	3595 (62)	20 (13)	89 (58)	< 0.001	3553 (61)	20 (13)	20 (13)	> 0.99
IB	1209 (21)	36 (23)	41 (27)		1189 (21)	36 (24)	35 (23)	
IIA	487 (8)	28 (18)	13 (8)		498 (9)	28 (18)	28 (19)	
IIB	336 (6)	32 (21)	10 (6)		329 (6)	32 (21)	31 (21)	
IIC	188 (3)	38 (25)	1 (1)		216 (4)	37 (24)	37 (25)	
**Body site (*n*** **(%))**								
Face/neck	453 (8)	14 (9)	6 (4)	0.48	460 (8)	14 (9)	12 (8)	0.65
Scalp	321 (6)	14 (9)	7 (5)		331 (6)	14 (9)	14 (9)	
Upper extremities	1333 (23)	28 (18)	34 (22)		1352 (23)	28 (18)	36 (24)	
Trunk	2303 (40)	59 (38)	64 (42)		2273 (39)	59 (39)	53 (35)	
Lower extremities	1400 (24)	39 (25)	43 (28)		1364 (24)	38 (25)	38 (25)	
**Morphological subtype (*n*** **(%))**								
Superficial spreading	4634 (80)	100 (64)	132 (86)	n/a	4587 (79)	100 (65)	94 (62)	n/a
Nodular	452 (8)	41 (27)	13 (8)		483 (8)	40 (26)	39 (26)	
Acral lentiginous	55 (1)	4 (3)	0 (0)		43 (1)	4 (3)	4 (3)	
Lentigo maligna melanoma	312 (5)	4 (3)	2 (1)		326 (6)	4 (3)	2 (1)	
Amelanotic melanoma	7 (0)	0 (0)	2 (1)		5 (0)	0 (0)	2 (1)	
Desmoplastic melanoma	35 (0)	0 (0)	1 (1)		30 (1)	0 (0)	2 (10)	
Unspecified	320 (6)	5 (3)	4 (3)		311 (5)	5 (3)	8 (5)	
**Surgical procedure (*n*** **(%))**								
Elliptical excision	n/a	137 (89)	137 (89)	n/a	n/a	136 (89)	134 (89)	n/a
Shave or punch biopsy	n/a	17 (11)	17 (11)		n/a	17 (11)	17 (11)	
**Sentinel lymph node biopsy (** ***n*** **(%))**								
Performed, overall	1893 (33)	72 (47)	49 (32)	0.01	1872 (32)	71 (46)	73 (48)	> 0.99
Not indicated, i.e. stage pT1a	2842 (49)	11 (7)	66 (43)		2823 (49)	11 (7)	16 (10)	
Eligible but not performed (stage pT1b or higher)	1102 (37)	71 (50)	41 (27)		1116 (38)	70 (50)	64 (42)	
**Time until stage IV recurrence**,** years (median (IQR**,** min-max range))**	1.8 (1.0–3.3, 0.3–5.3)	1.8 (1.0–3.3, 0.3–5.3)	n/a		1.8 (1.0–3.3, 0.3–5.3)	1.8 (1.0–3.3, 0.3–5.3)	n/a	
**Time until stage IV recurrence (n(%))**								
< 2 years	85 (55)	85 (55)	n/a		84 (55)	84 (55)	n/a	
2–5 years	66 (43)	66 (43)	n/a		66 (43)	66 (43)	n/a	
5–8 years	3 (2)	3 (2)	n/a		3 (2)	3 (2)	n/a	
> 8 years	0 (0)	0 (0)	n/a		0 (0)	0 (0)	n/a	
**Alive survival status at last follow-up (*n*** **(%))**	5029 (87)	44 (29)	143 (93)		4949 (86)	44 (29)	118 (78)	
**Follow-up duration until censoring or death**,** years (median (IQR))**	5.3 (4.0–6.6)	3.5 (1.9–5.4)	6.1 (4.8–7.3)		5.2 (4.0–6.6)	3.6 (1.9–5.4)	5.5 (3.7–7.0)	
**Follow-up duration until censoring or death**,** years (*n*** **(%))**								
< 2 years	297 (5)	42 (27)	3 (2)		309 (5)	42 (28)	10 (6)	
2–5 years	2348 (40)	66 (43)	39 (25)		2400 (42)	65 (43)	53 (35)	
5–8 years	3115 (54)	46 (30)	111 (72)		3008 (52)	46 (30)	87 (58)	
> 8 years	52 (1)	0 (0)	1 (1)		66 (1)	0 (0)	1 (1)	
**Disease progression after initial follow-up period (*n*** **(%))**								
Stage III	n/a	n/a	5 (3)		n/a	n/a	11 (7)	
Stage IV	n/a	n/a	1 (1)		n/a	n/a	0 (0)	

By matching on stage, multiple variables were more comparable between cases and the stage-matched controls, compared to the randomly selected controls (Table 1). In addition to Breslow thickness and ulceration, age distribution and sentinel lymph node biopsy status were balanced between cases and stage-matched controls. While most of all randomly selected controls were not eligible for a sentinel lymph node biopsy (66/154, 43%), due to their stage (pT1a), almost half of the stage-matched controls (74/153, 48%) underwent a sentinel lymph node biopsy. Body site distribution remained comparable between cases and stage-matched controls.

## Discussion

The D-ESMEL study was designed to identify new prognostic factors in addition to staging variables. While the original design of the validation cohort facilitated to build prognostic models including both known histopathological factors and new molecular factors, the nested case-control design with randomly selected controls lacked statistical power to perform stage-specific analyses and detect small differences within stage II. Therefore, we collected new stage-matched controls. This resulted in a nested case-control study that now includes 153 case-control sets matched on stage (IA, IB, IIA, IIB, IIC) based on a source cohort of 5,785 stage I/II melanoma patients in the Netherlands between 2016 and 2020. The extension of the D-ESMEL study now includes 97 stage II controls compared to 24 of the original design. These controls reflect much better the biological variability among patients without metastases in the total population.

With this extension, the D-ESMEL study will facilitate more in-depth analyses in stage II, providing sufficient statistical power to detect subtle differences in prognostic factors that were not detectable with the original study design. Sufficient statistical power and variability in this group are essential for developing accurate and stable prognostic models that are applicable in clinical practice. This is particularly relevant since stage IIC melanoma patients have a poorer prognosis than stage IIIA patients (i.e. sentinel lymph node metastasis < 1 mm). Therefore, clinical trials with adjuvant immunotherapy were conducted among stage II melanoma patients [7–9]. However, the absolute benefit across all stage II patients was low, with 5% to 6% reduction in the risk of developing distant metastasis [7, 8, 10]. Toxicity of immunotherapy should not be underestimated, as it can lead to lifelong adverse effects, such as adrenal insufficiency and diabetes. To prevent overtreatment of stage II melanoma patients who will never progress, accurate prognostic models are required. The NivoMela trial (NCT04309409) utilizes a gene expression profile for enhanced risk stratification in stage II melanoma. Although this strategy holds the potential to select patients more accurately, it should be carefully assessed whether these biomarkers stratify the patients more accurately than clinico-pathological factors alone. The original D-ESMEL study already facilitated the framework to analyze whether tissue-based biomarkers have prognostic value in addition to clinical staging in a population-based sample of stage I and II melanoma patients. The extension of the D-ESMEL study now facilitates population-based stage II subgroup analyses of prognostic tissue-based biomarkers in melanoma, supporting the development of more accurate and clinically applicable prognostic models for these patients.
